# Photobiomodulation attenuates oligodendrocyte dysfunction and prevents adverse neurological consequences in a rat model of early life adversity

**DOI:** 10.7150/thno.78777

**Published:** 2023-01-16

**Authors:** Zhihai Huang, Yulan Zhang, Xiaohui Ma, Yu Feng, Xuemei Zong, J. Dedrick Jordan, Quanguang Zhang

**Affiliations:** Department of Neurology, Louisiana State University Health Sciences Center, Shreveport, LA, 1501 Kings Highway, LA 71103 USA.

**Keywords:** Early life adversity (ELA), Photobiomodulation (PBM), Cognition, Depression, Oligodendrocyte

## Abstract

**Rationale:** Adverse experiences in early life including abuse, trauma and neglect, have been linked to poor physical and mental health outcomes. Emerging evidence implies that those who experienced early life adversity (ELA) are more likely to develop cognitive dysfunction and depressive-like symptoms in adulthood. The molecular mechanisms responsible for the negative consequences of ELA, however, remain unclear. In the absence of effective management options, anticipatory guidance is the mainstay of ELA prevention. Furthermore, there is no available treatment that prevents or alleviates the neurologic sequelae of ELA, especially traumatic stress. Hence, the present study aims to investigate the mechanisms for these associations and evaluate whether photobiomodulation (PBM), a non-invasive therapeutic procedure, can prevent the negative cognitive and behavioral manifestations of ELA in later life.

**Methods:** ELA was induced by repeated inescapable electric foot shock of rats from postnatal day 21 to 26. On the day immediately following the last foot shock, 2-min daily PBM treatment was applied transcranially for 7 consecutive days. Cognitive dysfunction and depression-like behaviors were measured by a battery of behavioral tests in adulthood. Subsequently, oligodendrocyte progenitor cells (OPCs) differentiation, the proliferation and apoptosis of oligodendrocyte lineage cells (OLs), mature oligodendrocyte, myelinating oligodendrocyte, the level of oxidative damage, reactive oxygen species (ROS) and total antioxidant capacity were measured and analyzed using immunofluorescence staining, capillary-based immunoassay (ProteinSimple®) and antioxidant assay kit.

**Results:** The rats exposed to ELA exhibited obvious oligodendrocyte dysfunction, including a reduction in OPCs differentiation, diminished generation and survival of OLs, decreased OLs, and decreased matured oligodendrocyte. Furthermore, a deficit in myelinating oligodendrocytes was observed, in conjunction with an imbalance in redox homeostasis and accumulated oxidative damage. These alternations were concomitant with cognitive dysfunction and depression-like behaviors. Importantly, we found that early PBM treatment largely prevented these pathologies and reversed the neurologic sequelae resulting from ELA.

**Conclusions:** Collectively, these findings provide new insights into the mechanism by which ELA affects neurological outcomes. Moreover, our findings support that PBM may be a promising strategy to prevent ELA-induced neurologic sequelae that develops later in life.

## Introduction

Early life is a crucial period for brain development, and the developing brain is particularly sensitive to environmental events 1, 2. During this time, exposure to adverse experiences such as physical abuse, traumatic events, and neglect can be detrimental to psychological and physical health 3, 4. A vast body of work supports the idea that early life adversity (ELA) is a risk factor for developing various mental illnesses in adolescence and adulthood, including depression, bipolar and anxiety disorders 5-8. ELA also may negatively affect the development of cognitive functions 9-12. Many cross-sectional surveys have pointed out that individuals who experience ELA exhibit greater vulnerability to psychological illnesses and show a deficit in cognitive function 13-16. A variety of efforts have been made to prevent ELA, such as mitigating childhood poverty and improving parental care 4, 17. Nevertheless, many forms of ELAs, particularly traumatic events and abuse, continue to impact large numbers of children worldwide 18, 19. Therefore, early management following exposure to ELA is of significant importance as it may lead to a reduction or possibly prevent these adverse consequences in later life.

In the central nervous system, oligodendrocyte lineage cells (OLs), including oligodendrocyte progenitor cells (OPCs) and mature oligodendrocytes, have a variety of functions 20, 21. These cells are responsible for the formation of the myelin sheath, enabling the rapid and efficient transmission of electrical impulses along axons, thereby maintaining axonal integrity 21-23. Oligodendrocyte and myelin dysfunction have been linked to multiple psychiatric and neurodegenerative disorders 24-26. Indeed, oligodendrocytes and myelin in the hippocampus are extremely sensitive to stressful experiences 27-29, and emerging evidence demonstrates that deficits in oligodendrogenesis and myelination may be a major contributor in the pathogenesis of several mental illnesses such as depression and anxiety disorders 30-32. Abnormal OPCs and myelin have been frequently been reported in the brains of individuals with depression and stress-related disorders, as well as in experimental animal models of these conditions 31, 33-36. Of note, in rodent models, ablation of OPCs or inhibition of oligodendrocyte maturation resulted in myelin loss accompanied by depressive-like behaviors 37, 38, and these effects can be reversed by restoring OPCs or administering clemastine, a pro-remyelinating antimuscarinic drug 37, 39. These findings suggest oligodendrocytes may be a potential target for the treatment of depression and stress-related disorders. Furthermore, recent work has shown that well-functioning oligodendrocyte and myelin were required for memory consolidation and cognitive flexibility 40-42. Spatial learning promoted oligodendrogenesis and myelination in the hippocampus, while inhibition of these processes can impair spatial memory in young animals 40, 41. More importantly, enhancing myelination or promoting oligodendroglial differentiation rescued memory decline during aging 41. Intriguingly, animals exposed to ELA also exhibited a deficiency in oligodendrogenesis, which was positively associated with emotional and cognitive disorders in adulthood 29, 43. Therefore, the deficiency in oligodendrogenesis and myelin loss could be common pathological processes underlying ELA-induced emotional and cognitive disorders.

As a non-invasive procedure, photobiomodulation (PBM) has been receiving increased interest in the treatment of various neurological and psychological disorders, such as anxiety and depression 44-46, stress-related disorder 47, 48, stroke 49-51, and Alzheimer's disease 52-54. Although the excitation of mitochondrial cytochrome c oxidase (CCO) was once thought to be the primary mechanism underlying the action of PBM, its mechanisms of action have recently been identified to involve multiple physiological processes and signals. Intriguingly, this non-invasive therapy has been extensively proven to modulate the redox metabolism, including alleviating oxidative damage and improving enzymatic antioxidant activity 55, 56. Hence, the regulation of redox metabolism could be a crucial and common mechanism of PBM for a variety of disorders. Indeed, our previous studies have shown that transcranial PBM can effectively prevent stress-related disorders in adult rats, including the mitigation of cognitive deficit, anxiety, and depression-like behaviors 48. At tissue and cell levels, PBM modulates various molecular events, including enhanced mitochondrial enzymes, decreased neuroinflammation, neurotrophic factors secretion, and upregulated energy substrate synthesis 57-60. Notably, in animal models of demyelinating diseases, PMB also facilitated OPCs proliferation and myelin repair, thereby promoting neuroplasticity and consequently functional recovery 61, 62. These results implied that PBM may contribute to the recovery of multiple brain disorders by modulating oligodendrogenesis and myelination.

Here, using a rat model of ELA, we investigated the therapeutic benefits of PBM, a non-invasive physical therapy, when administered early after ELA exposure. The results from the current study suggests that exposure to ELA leads to cognitive disorder and depressive-like behaviors in adulthood. Mechanistically, ELA resulted in decreased proliferation and differentiation of OPCs, with higher apoptosis levels. Similarly, myelin loss and elevated oxidative damage levels were found. More significantly, these alternations can be prevented by early PBM treatment after ELA. Together, these results demonstrate that PBM may be an effective therapeutic strategy to prevent the ELA-induced negative consequences later in life.

## Material and methods

### Animals and experimental design

Sprague-Dawley rat pups of both sexes were bred in our laboratory. Briefly, the pups were kept with their mothers until weaning (postnatal day 21), and then housed in same-sex cages with littermates. The animals were randomly divided into three groups: 1) control group, 2) control + PBM 3) foot shock group, and 4) foot shock + PBM treatment group, with 5 - 7 male and female pups in each group. The pups were group-housed in a temperature- and humidity-controlled room, under a 12-hour dark-light cycle (lights on 6 a.m-6 p.m.), with ad libitum access to water and food. All procedures were performed in compliance with the National Institutes of Health guidelines and were approved by the Institutional Animal Care and Use Committee (Louisiana State University Health Sciences Center at Shreveport). All efforts have been made to minimize animal suffering and the number of animals used.

### Repeated inescapable electric foot shock

ELA was induced by repeated inescapable electric foot shock, as described in previous studies 63-65. Briefly, between postnatal day 21-26, the rats were placed in a closed, dark electric box with electrified bottom and received 0.5 mA electric foot shock daily. Each foot shock lasted for 6 sec, repeated 20 times with a 30 sec interval, and was repeated for two trials per day. After the last foot shock stimulation, rats were remained in the box for 5 min and then were returned to their cage. The animals in non-foot shock control group were located in the box for the same period of time, but without foot shock. This procedure was performed during the same time period each morning.

### PBM treatment

The PBM treatment was performed as described in our previous work 48. In brief, from the day after the last foot shock, rats were subjected to daily 2-min PBM treatment (808 nm laser, continuous wave, 25 mW/cm^2^, 3 J/day) for 7 consecutive days. A diode laser (model 808M100, Dragon Lasers, Jilin province, China) was used to deliver PBM, and focus on the beam into a 1.5 cm round spot covering the shaved scalp.

### Behavioral testing

Before the behavior tests, rats were placed in the testing room for at least one hour and left undisturbed in a quiet setting. All behavioral tests were conducted during the light phase, and “ANY-maze” software (Stoelting Co. Wood Dale, IL) was used to record and analyzed the data. All tests and data analysis were conducted by independent researchers blinded to the experiment design. The test field was cleaned between each trial using 70% ethanol to avoid odoriferous cues.

### Barnes maze task

The Barnes Maze task was used to assess spatial learning and memory in animals 66. Briefly, this task consisted of three days of training trials and one probe trial. During the training trials, animals were allowed to explore freely for up to 3 min, and their motion tracks were recorded with an overhead camera connected with ANY-maze video tracking software (Stoelting, Wood Dale, IL, USA). The latency time to find the hidden chamber and a total number of errors were recorded and analyzed. On the probe day, the time spent of the animals in the target quadrant where the target hole was located and the number of the target hole entrances were recorded.

### Novel object recognition test

The novel object recognition test was applied to measure the recognition memory 67. On the first day, the rats were placed in an empty box (height: 40 cm; width: 50 cm; length: 50 cm), two identical objects were placed on the floor of the box at equal distances and the rats were allowed to explore both objects for 5 minutes. On the second day (test day), one of the objects was replaced with a new object of the same height, and the animals were given 5 min to explore. The traces of the animal and the time spent exploring each object were recorded and analyzed. The following formula was used to determine the discrimination index: Discrimination index = (time spent on novel object/ total time spent on both exploring objects)×100%.

### Forced swimming test

The forced swimming test is a well-validated and commonly performed test to evaluate depressive-like behavior 48. In brief, the rats were tested in a circular tank (30 × 37 × 56 cm). The tank was filled with 36 cm of water and allowed the animals to swim for 5 min. Immobilization, defined as a rat not moving its four claws for at least one second, was recorded.

### Tail suspension test

The tail suspension test was used to test depressive-like behavior 68. In brief, the rat's tail was suspended in the middle of the suspension box 50 cm above the ground. The test was performed over a 5-min session, and the immobile time was recorded.

### Elevated plus maze

As described in previous studies 68, the elevated plus-maze was used to evaluate anxious-like behavior. Briefly, two oppositely positioned open arms and closed arms, and a central zone was included in the apparatus elevated plus-maze, which was 50 cm above the floor. The two oppositely positioned closed arms were surrounded by 50-cm-high walls. The animals were placed in the center of the maze and allowed them to freely explore for 5 min. The time spent in the open arms and the number of entries into the open arms were recorded.

### Body weight

Animal body weight was recorded before the induction of foot shock and subsequently monitored weekly. The body weight of each rat was measured and normalized as 100 %.

### Brain collection and tissue preparation

Brain collection was performed after behavioral testing. Briefly, after sacrifice, the rat's brain was rapidly collected. One side of the brain was post-fixed with 4% hydrated paraformaldehyde (Thermo Fisher) for tissue sections. The hippocampal tissue was stripped from the other hemisphere. Brain sections (25 μm) were prepared using a Leica Rm2155 microtome and stored in stock solution (FD NeuroTechnologies, Inc., Columbia, MD, USA) for immunofluorescence staining. A motor-driven Teflon homogenizer with a 400 μL ice-cold mixture of homogenization buffer, protease, and phosphatase inhibitors was used to prepare protein from the hippocampus. To get total protein or subcellular fractions, the homogenates were centrifuged at the appropriate speed and time.

### Capillary-based immunoassay via protein simple®

ProteinSimple® capillary-based immunoassay (The Jesss system, ProteinSimple®, San Jose, CA) was performed as previously reported 69. The technology is an automated capillary size separation and nanoimmunoassay system that incorporates and automates the entire protein separation and detection process using homemade antigens. According to the manufacturer's protocol, primary antibodies were diluted targeting the following proteins: PDGFRα (1:50, Cell Signaling Technology), MBP (1:50, Abcam), Olig2 (1:50, R&D Systems). Antibody targets were detected with HRP-conjugated secondary antibodies. Digital images were analyzed using Compass for SW software (V6.1.0, Protein Simple) and quantified data of detected proteins were reported.

### Immunofluorescence staining

Immunofluorescence staining was conducted as previously described in our laboratory 68. Briefly, coronal brain slices were first incubated for 5 h with 0.4% Triton X-100 (Thermo Fisher), followed by 10% normal donkey serum for another 1 h at room temperature. After that, the brain slices were incubated with suitable primary antibodies in 0.1 % Triton X-100 overnight at 4 °C. In this study, the following antibodies were employed: MBP, Olig2, PDGFRα, CC1, Ki67, and 4HNE, as shown in **Table S1**. After incubation with primary antibodies, brain slices were washed four times with 0.1% Triton X-100 and then incubated with appropriate fluorescently labeled secondary antibodies (647/568/488, Thermo Fisher Scientific) at room temperature for 1 h. After incubation with secondary antibodies, brain slices were then washed and incubated with DAPI (Vector Laboratories, California). Fluorescence images were captured using the Zeiss Axio Observer. Z1 Motorized Fluorescence Microscope. Image J software was used to analyze all of the fluorescence images.

### Measurement of ROS level

The level of ROS was detected by dihydroethidium (DHE) probe. DHE (AnaSpec) was added to the hippocampal homogenate to a final concentration of 10 mM. The mixture was incubated for 10 min at RT in the dark. The fluorescence intensity was measured at 485 nm excitation/590 nm emission.

### Total antioxidant capacity assay

Total antioxidant capacity was measured by an antioxidant assay kit (709001; Cayman Chemical) according to the instructions of the vendor, as previously described 70. Briefly, Pre-diluted samples (10 μL) in assay buffer were added to the designated wells with 10 KL of high iron myoglobin and 150 μL of chromogen. After adding 40 μL of hydrogen peroxide working solution, the plate was sealed and incubated on a shaker for 5 minutes. Then, in accordance with the assay protocol, the absorbance at 750 nm was measured on a spectrophotometer, and the standard was plotted as a function of the final trolox concentration (mM). The trolox standard curve was then used to calculate each sample's antioxidant capacity, and the results were presented as a percentage change from the control.

### TUNEL assay

The TUNEL assay was performed to detect the apoptosis of OPCs. A Click-iT® Plus TUNEL assay kit (Thermo Fisher Scientific) was used to label cellular apoptosis following the manufacturer's instructions.

### Statistical analysis

GraphPad Prism Software was used to perform statistical analyses. One way analysis of variance (ANOVA) or two-way ANOVA followed by Newman-Keuls multiple comparison post test was performed to analyze all dependent variables. For comparisons between two groups, data were analyzed by the student's t-test. All data were presented as mean ± SE. A value of P < 0.05 was considered significant for all statistical tests.

## Results

### ELA induces transient slowdown in weight gain

**Figure 2:**
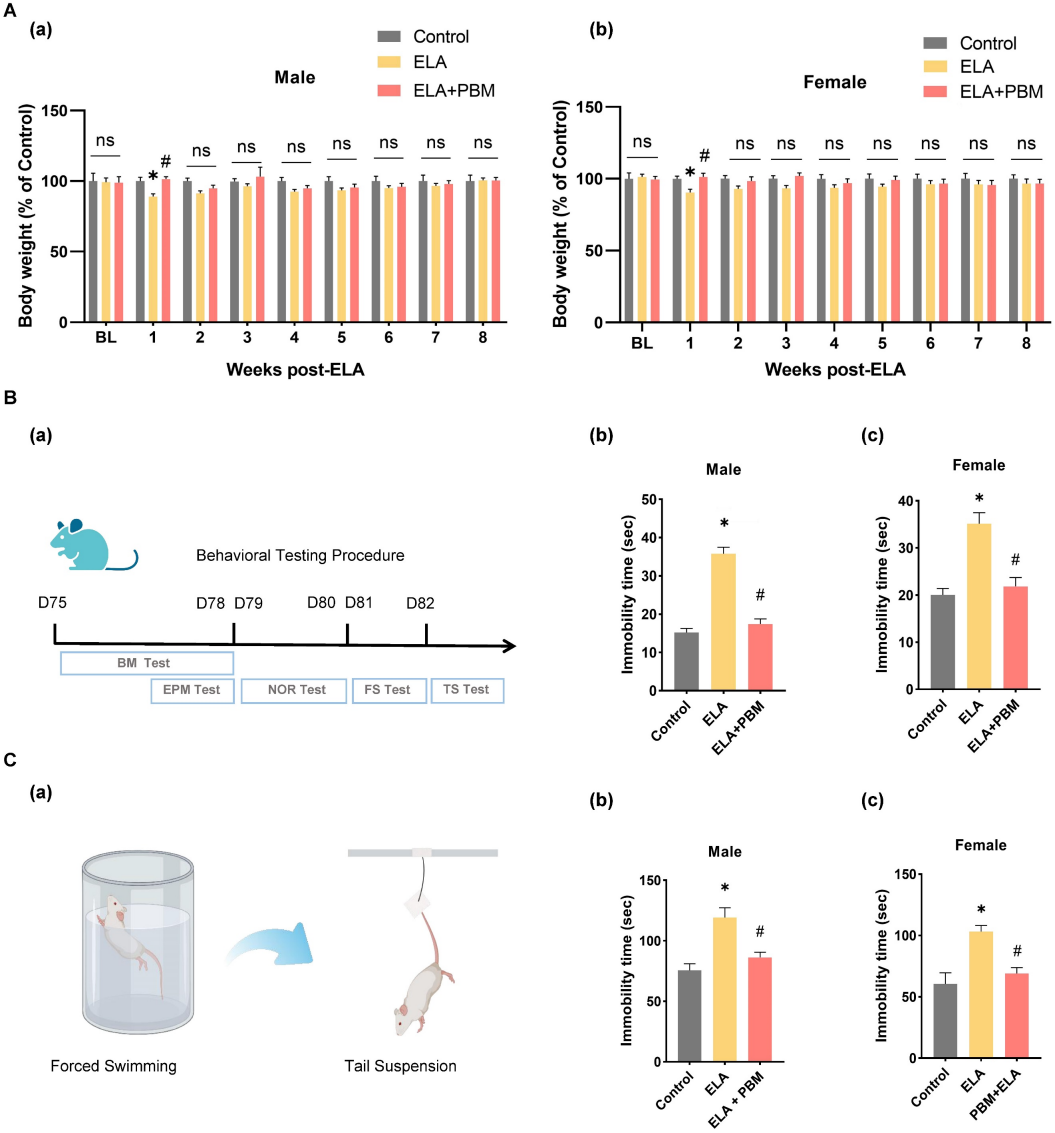
** Early PBM treatment prevents alternations in body weight and ELA-induced depressive-like behaviors. A** No significant difference in BL (baseline) body weight among all groups following ELA. **B (a)** Schematic diagram displaying the time course of behavioral testing. **B (b, c)** The forced swimming test and tail suspension test were conducted to measure depressive-like behaviors, in the forced swimming test, ELA-exposured rats exhibited dramatically longer immobility time than the control group, which can be reversed by early PBM treatment. **C (a)** Schematic illustrating the forced swimming test and tail suspension test. **C (b, c)** PBM-treated ELA rats also exhibited less immobility time in tail suspension test. All data are presented as mean ± SE (n = 5-7). * P < 0.05 versus Control-group; ^#^ P < 0.05 versus ELA-group.

Previous studies reported that ELA may cause delayed physical development 71. Therefore, we measured the body weight of the animals weekly after the induction of ELA. As shown in **Figure 2A (a-b)**, at baseline, no significant differences in body weight were observed among all groups. Notably, in the first week after ELA, rats in the ELA group exhibited a significant reduction in body weight gain (Male, *P* = 0.0300, Control vs. ELA; Female, *P* = 0.0414, Control vs. ELA). Subsequently, we explored whether PBM could prevent ELA-induced temporary reductions in weight growth. We found that early PBM intervention prevented weight loss in both genders at week 1 post ELA (Male, *P* = 0.0172, ELA+PBM vs. ELA; Female, *P* = 0.0293, ELA+PBM vs. ELA). Nevertheless, from week 2 to 8, these alternations were small or not statistically significant from week 2 to 8 among all groups.

### Early PBM treatment prevents ELA-induced depressive-like behaviors and comorbidity

To test the effect of PBM on ELA-induced depressive-like symptoms, we performed the forced swim test and tail suspension test. In the forced swim test (**Figure 2B (b-c)**), animals exposed to ELA showed longer immobility time, an indicator of depression-like behavior (Male, *P* < 0.001, Control vs. ELA; Female, *P* < 0.0001, Control vs. ELA). Strikingly, animals in the PBM group showed significantly less immobility time, indicating that depression-like behavior was mitigated (Male, *P* < 0.0010, ELA+PBM vs. ELA; Female, *P* < 0.0001, ELA+PBM vs. ELA). To further validate the efficacy of PBM, we performed the tail suspension test. As illustrated in **Figure 2C (b-c)**, animals in the ELA group also exhibited longer immobility times in this test (Male, *P* < 0.0010, Control vs. ELA; Female, *P* < 0.0001, Control vs. ELA), which could be reversed by early PBM treatment (Male, *P* = 0.0070, ELA+PBM vs. ELA; Female, *P* < 0.0001, ELA+PBM vs. ELA).

**Figure 3:**
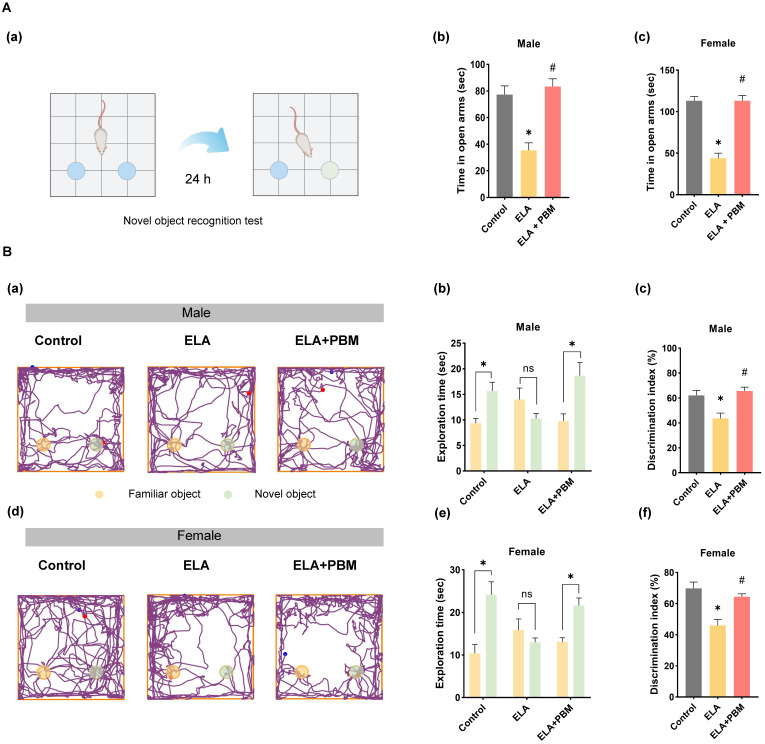
** Early PBM treatment prevents ELA-induced cognitive deficits and comorbidity. A (a)** Schematic diagram of the novel object recognition test. The novel object recognition test was conducted to measure the recognition memory. **A (b, c)** The elevated plus maze text was performed to measure anxious-like behavior, PBM-treated ELA rats spent significantly more time in the open arms than their untreated counterparts. **B (a, d**) The representative tracking plots on the novel object recognition test. Between all groups, the time spent on each object and discrimination index was calculated and statistically compared **(b-c, e-f)**. ELA rats spent significantly less time exploring the novel object, while early PBM treatment reserved these deficits. All data are presented as mean ± SE (n = 5-7). * P < 0.05 versus Control-group; ^#^ P < 0.05 versus ELA-group.

Anxiety is a common comorbidity in patients with depression. It has been reported that about 85% of patients with depression exhibit significant anxiety, and depression remission contributes to improved anxiety-like symptoms 72. Therefore, to test the effect of PBM on anxiety-like symptoms, the elevated plus maze test was performed. As shown in **Figure 3A (b and c)**, compared to animals in the control group, animals exposed to ELA spent significantly less time exploring the open arms (Male, P = 0.0090, Control vs. ELA; Female, P < 0.0010, Control vs. ELA). Intriguingly, this behavioral abnormality was reversed in animals treated with PBM, as evidenced by longer exploration times in the open arms (Male, P = 0.0030, ELA+PBM vs. ELA; Female, P < 0.0010, ELA+PBM vs. ELA).

### Early PBM treatment prevents ELA-induced cognitive dysfunction in adulthood

Using the Barnes maze task and novel object recognition test, we evaluated the effects of ELA on cognitive performance of animals in adulthood. The novel object recognition test was first applied to test recognition memory. This test involves recollection and familiarity, and it evaluates the ability of rodent animals to discriminate a novel object from a familiar object. As illustrated in **Figure 3A (a-e)**, we observed that ELA-exposed animals exhibited recognition memory impairment, as evidenced by a remarkable reduction in the time spent exploring the novel object (Male, *P* = 0.0107, Control vs. ELA; Female, *P* = 0.0009, Control vs. ELA). Intriguingly, further analysis revealed that early PBM intervention substantially reversed these deficits (Male, *P* = 0.0044, ELA+PBM vs. ELA; Female, *P* = 0.0116, ELA+PBM vs. ELA).

Next, the Barnes maze task was conducted to assess spatial learning and memory, and striking sex differences were observed. Specifically, compared to animals in the control group, male animals exposed to ELA demonstrated substantial memory learning deficits in the day 2 acquisition phase (*P* = 0.0430, ELA vs. Control) (**Figure S1B (a))**, whereas female animals did not show these deficits (**Figure S1B (c))**. To further investigate the influences of ELA on spatial memory, we conducted a probe test following the last training day. As shown in **Figure S1B (b)** and **Figure S1A (d)**, both male and female animals in the ELA group spent significantly less time in the target quadrant (Male, *P* = 0.0190, Control vs. ELA; Female, *P* = 0.0216, Control vs. ELA), and early PBM administration partially reversed this memory deficit but was not statistically significant (Male, *P* = 0.4596, ELA+PBM vs. ELA; Female, *P* = 0.1645, ELA+PBM vs. ELA).

### ELA diminishes the differentiation of OPCs into OLs and can be reversed by early PBM treatment

**Figure 4:**
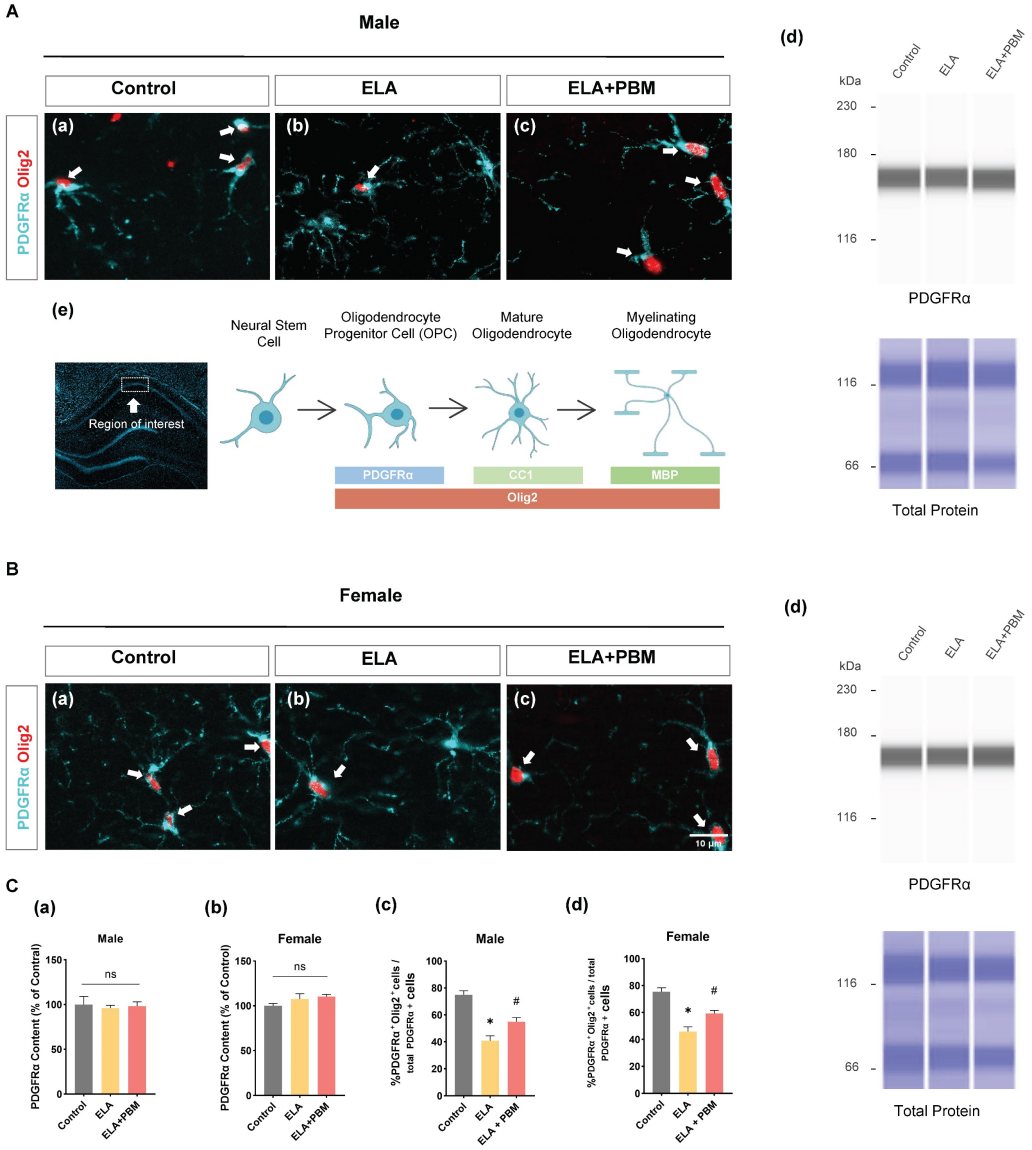
** ELA diminishes the differentiation of OPCs into OLs and can be reversed by early PBM treatment. A (a-c) and B (a-c)** Representative immunofluorescence staining for - OLs (Olig2^+^, red) - labeled PDGFRα^+^ (blue) in CA1 region of hippocampus. **A (d) and B (d)** Representative synthetic protein bands of PDGFRα in the hippocampus, corresponding peak areas were normalized with the level of total protein. **A (e)** The depicted area was imaged in the hippocampal CA1 region, graphical representation of the different oligodendrocyte lineage stages and respective markers used in this study. **C (a, b)** Results of quantitative analysis of PDGFRα protein levels. **C (c, d)** Percentage of Olig2+- labeled OPCs (Olig2+ PDGFRα^+^ cells) in the total PDGFRα^+^ cells. ELA-exposured rats exhibited a dramatic reduction in the differentiation of OPCs into OLs, which was reversed by early PBM treatment. Scale bar = 10 µm. All data are presented as mean ± SE (n = 5-7). * P < 0.05 versus Control-group; ^#^ P < 0.05 versus ELA-group.

To determine whether ELA would result in reduced OPCs, we examined the expression of PDGFRα (a marker of OPCs) by ProteinSimple® capillary electrophoresis system. Statistically significant differences, however, were not observed across groups, as shown in **Figure 4C (a-b)**. Additionally, brain sections were co-stained for PDGFRα and Olig2 (a marker of OLs), to evaluate the differentiation of OPCs into OLs in the hippocampus CA1 area. As quantitated in **Figure 4C (c-d)**, a decreased proportion of PDGFRα^+^/Olig2^+^ cells was observed in ELA exposed animals (Male, *P* < 0.0010, Control vs. ELA; Female, *P* < 0.0010, Control vs. ELA). This finding corresponds with previous observations that stress in early life results in the impaired differentiation ability of OPCs in adults 43. We next investigated if early PBM treatment could rescue ELA-induced OPCs dysfunction. Remarkably, the PBM-treated ELA animals showed greater OPCs differentiation in comparison to the ELA group, despite there still being some differences between the control group and PBM group (Male, *P* = 0.0145, ELA+PBM vs. ELA; Female, *P* = 0.0134, ELA+PBM vs. ELA).

### Early PBM treatment prevents ELA-induced OLs apoptosis and reduction in mature oligodendrocytes

**Figure 5:**
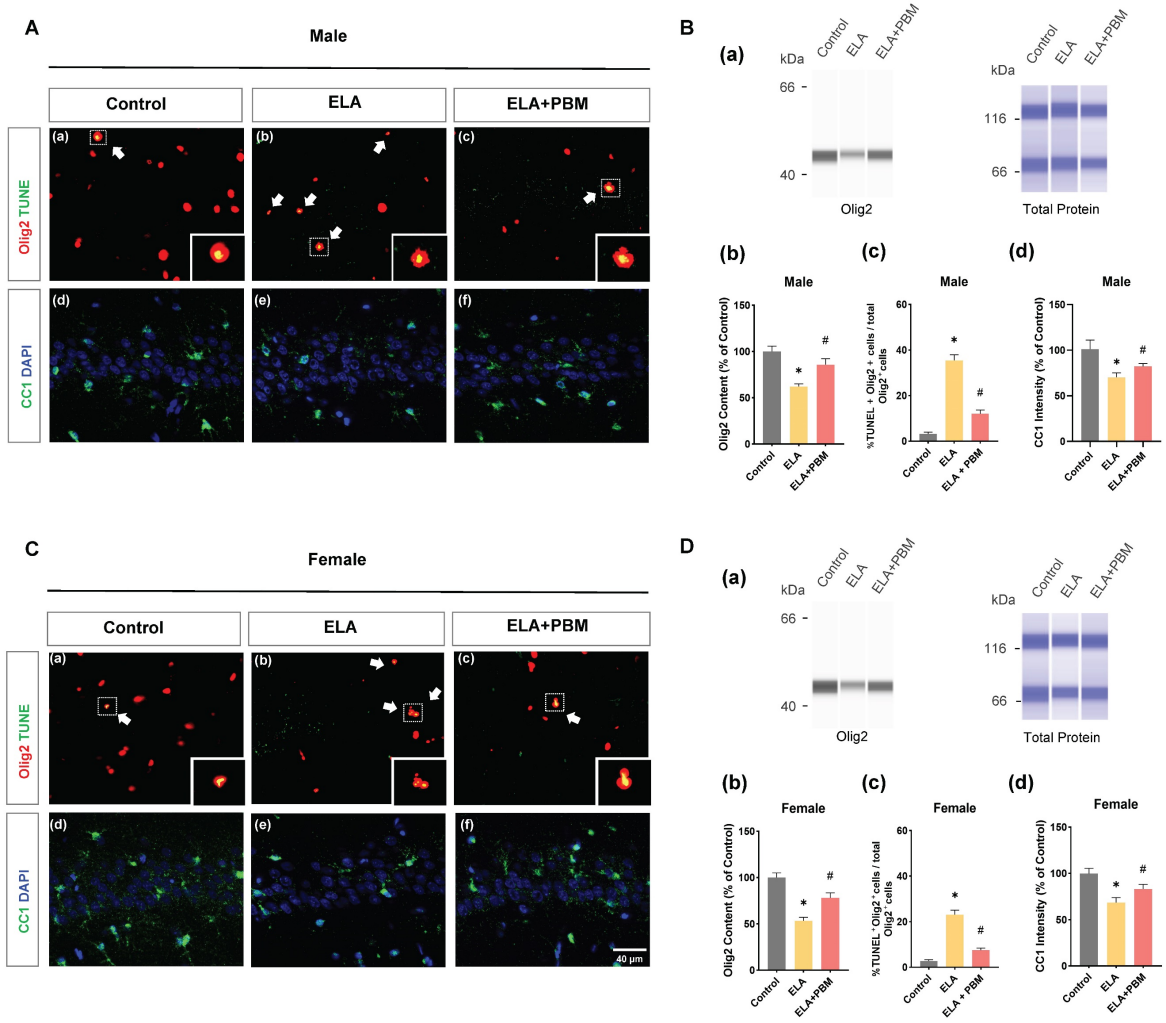
** Early PBM treatment prevents ELA-induced OLs apoptosis and reduction in mature oligodendrocytes. A (a-c) and C (a-c)** Representative immunofluorescence staining for TUNEL^+^ (green) - labeled OLs (Olig2^+^, red) in CA1 region of the hippocampus, small images exhibit a representative single cell from each group. **B (a) and D (a)** Representative synthetic protein bands of Olig2 in the hippocampus, corresponding peak areas were normalized with the level of total protein. **B (b) and D (b)** Results of quantitative analysis of Olig2 protein levels. **B (c) and D (c)** Percentage of TUNEL^+^ - labeled OLs (TUNEL^+^ Olig2^+^ cells) in the total Olig2^+^ population. **A (d-f) and C (d-f)** Representative immunofluorescence staining for mature oligodendrocyte (CC1, green) with DAPI. **B (d) and D (d)** Quantification of CC1^+^ cells. ELA-exposured rats exhibited a marked increase in the proportion of oligodendrocyte apoptosis, accompanied by reductions in Olig2 protein content and mature oligodendrocyte (CC1), which was reversed by early PBM treatment. Scale bar = 40 µm. All data are presented as mean ± SE (n = 5-7). * P < 0.05 versus Control-group; ^#^ P < 0.05 versus ELA-group.

Chronic or persistent stress has been demonstrated to induce oligodendroglial apoptosis 73. To investigate if exposure to ELA would result in accelerated oligodendroglial apoptosis, we performed double immunostaining with Olig2 (a marker of OLs) and TUNEL in the hippocampus CA1 area **(Figure 5A (a-c) and C (a-c)).** As quantified in **Figure 5B (c) and D (c)**, ELA - exposed rats exhibited increased OLs apoptosis, which seemed more salient in male animals (Male, *P* < 0.0001, Control vs. ELA; Female, *P* < 0.0001, Control vs. ELA). Of note, we also found that animals treated with PBM showed a significant reduction in the proportion of TUNEL^+^/Olig2^+^ cells (Male, *P* < 0.0001, ELA+PBM vs. ELA; Female, *P* < 0.0001, ELA+PBM vs. ELA). Next, protein level of Olig2 was evaluated using ProteinSimple capillary immunoassay. As quantified in **Figure 5B (b) and D (b)**, a marked reduction in Olig2 content was observed following ELA (Male, *P* = 0.0054, Control vs. ELA; Female, *P* = 0.0012, Control vs. ELA), while early PBM treatment ameliorated this downregulation (Male, *P* = 0.0436, ELA+PBM vs. ELA; Female, *P* = 0.0270, ELA+PBM vs. ELA). Furthermore, there was no appreciable cell death in OPCs (**Figure S2**), suggesting that ELA did not compromise the survival of the OPCs.

Next, we stained for CC1/DAPI to determine whether ELA could affect the maturation of oligodendrocytes (**Figure 5B (d) and D (d))**. As expected, a reduction in CC1 was observed in ELA-exposured rats (Male, *P* < 0.0010, Control vs. ELA; Female, *P* <0.0010, Control vs. ELA). In parallel, this change was mitigated by early PBM treatment, as indicated in enhanced CC1 intensity (Male, *P* = 0.0160, ELA+PBM vs. ELA; Female, *P* <0.0010, ELA+PBM vs. ELA).

### ELA leads to diminished proliferation of OLs, which can be rescued by early PBM treatment

**Figure 6:**
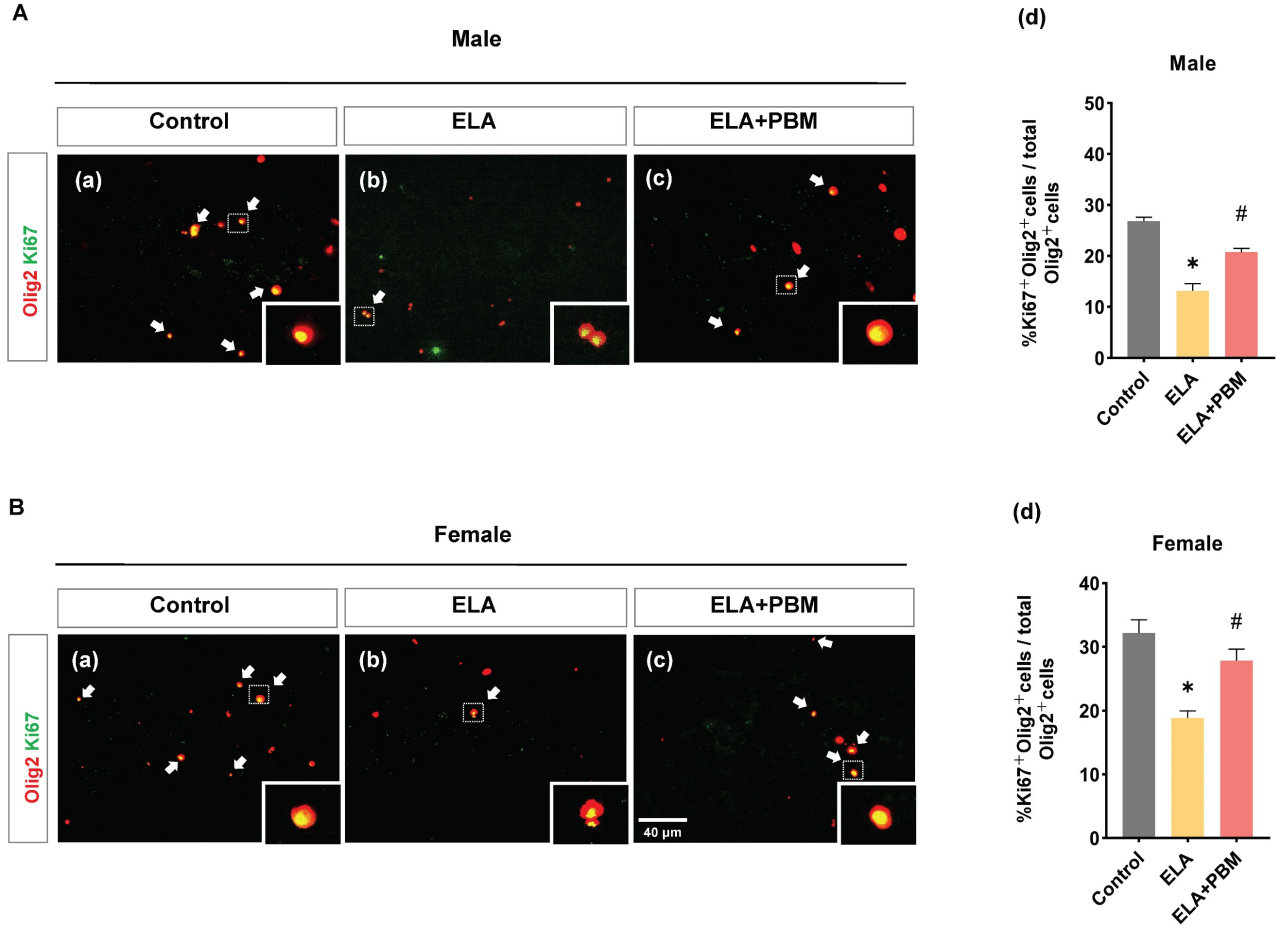
** ELA leads to diminished proliferation of OLs, which can be rescued by early PBM treatment. A (a-c) and B (a-c)** Representative immunofluorescence staining for Ki67^+^ (green) - labeled OLs (Olig2^+^, red) in CA1 region of the hippocampus, small images exhibit a representative single cell from each group. **(A) (d) and B (d)** Percentage of Ki67^+^ - labeled OLs (Ki67^+^ Olig2^+^ cells) in the total Olig2^+^ cells. ELA-exposured rats exhibited a dramatic decrease in the proportion of newly differentiated OLs, while PBM-treated ELA rats showed normal differentiation capacity. Scale bar = 40 µm. All data are presented as mean ± SE (n = 5-7). * P < 0.05 versus Control-group; ^#^ P < 0.05 versus ELA-group.

OLs are able to regenerate throughout the life, and are thought to positively impact the development and function of the CNS 74. Using double staining of Ki67, an endogenous marker of mitosis, and Olig2, we investigated whether exposure to ELA would lead to a reduction of self-renewal capacity of OLs. Strikingly, a remarkable reduction in proliferating OLs was observed in ELA-exposured rats (Male, *P* < 0.0001, Control vs. ELA; Female, *P* < 0.0001, Control vs. ELA), as evidenced by decreased proportion of Ki67^+^ /Olig2^+^ cells **(Figure 6A (d) and B (d))**. Intriguingly, this reduction was significantly ameliorated in rats treated with PBM (Male, *P* < 0.0001, ELA+PBM vs. ELA; Female, *P* = 0.0072, ELA+PBM vs. ELA).

### ELA leads to a reduction in myelinating oligodendrocytes, which can be partially mitigated by early PBM treatment

**Figure 8:**
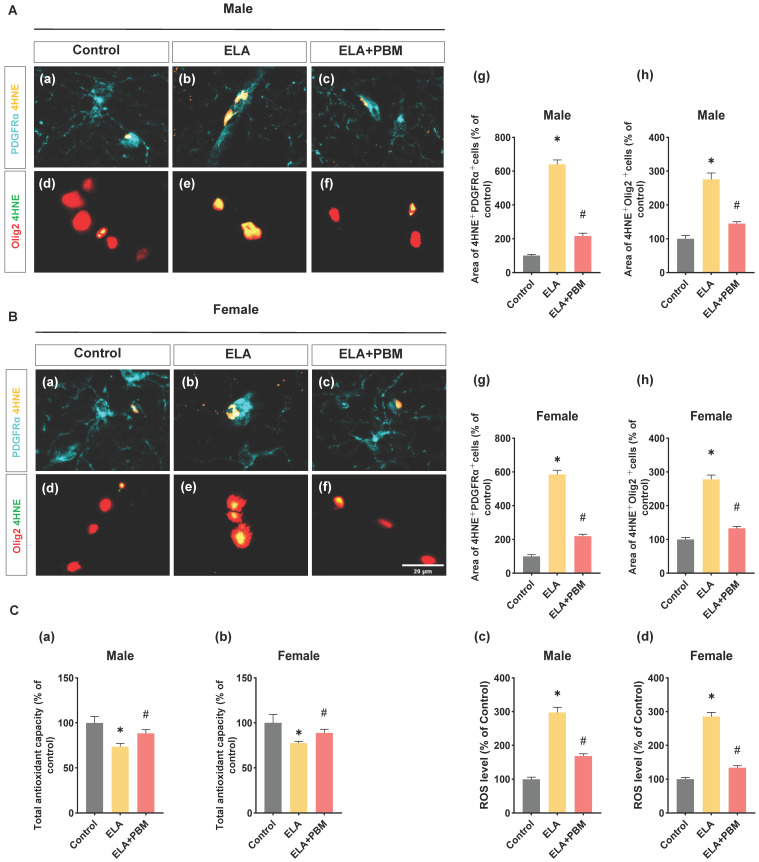
** Early PBM treatment mitigates ELA-induced chronic oxidative damage in oligodendrocyte. A (a-f) and B (a-f)** Representative immunofluorescence staining for 4HNE with PDGFRαand Olig2 in CA1 region of the hippocampus.** A (g) and B (g)** Fluorescent intensity of 4HNE was calculated by ImageJ analysis software and expressed as percentage changes versus respective control group. **A (h) and B (h)** Detection and quantification of relative levels of ROS in protein samples by fluorescence spectrophotometry. ROS and oxidative damage levels were significantly increased in animals exposed to ELA, which was attenuated by PBM treatment.** C (a, b)** Quantitative analysis of total antioxidant capacity by an antioxidant assay kit. ELA leads to compromised antioxidant capacity, and early PBM treatment largely reverses this deficiency. Scale bar = 20 µm. All data are presented as mean ± SE (n = 5-7). * P < 0.05 versus Control-group; ^#^ P < 0.05 versus ELA-group.

To investigate the impact of ELA on myelinating oligodendrocytes, capillary-based immunoassays were performed on hippocampal tissue, and MBP, a well-established marker labeling myelinating oligodendrocytes were used. As quantified in **Figure 8B (b)** and **Figure 8D (b)**, drastically reduced MBP protein expression was observed in the ELA group (Male, *P* = 0.0115, Control vs. ELA; Female, *P* = 0.0226, Control vs. ELA). Moreover, early PBM treatment partially attenuated the downregulation of MBP. The difference, however, was not significant (Male, *P* = 0.0880, ELA+PBM vs. ELA; Female, *P* = 0.1187, ELA+PBM vs. ELA). Notably, while there was no statistical difference between the PBM-treated and ELA groups, the PBM group did not differ from the control group either (Male, *P* = 0.2717, ELA+PBM vs. Control; Female, *P* = 0.4208, ELA+PBM vs. Control), suggesting that PBM may still partially mitigate the reduction in MBP following exposure to ELA. Consistently, representative images of immunofluorescence staining show the structural alterations of MBP (**Figure 8A (a-f) and Figure 8C (a-f)**).

### Early PBM treatment mitigates ELA-induced redox imbalance and oxidative damage

Oligodendrocytes, including OPCs, are particularly vulnerable to oxidative damage 75. Under conditions of oxidative stress, differentiation of OPC and oligodendrocytes maturation can be disrupted, and compared to mature oligodendrocytes, OPCs have increased susceptibility to oxidative damage 76, 77. Therefore, to gain more insight into the mechanisms through which PBM alleviates oligodendrocyte dysfunction induced by ELA, we conducted double staining for 4-HNE (a marker of oxidative damage) and PDGFRαor Olig2. As qualified in **Figure 8A (g) and B (g)**, the rats exposed to ELA showed higher 4HNE+PDGFRα area, suggesting that ELA induced long-term oxidative damage in OPCs (Male, *P* < 0.0001, Control vs. ELA; Female, *P* < 0.0001, Control vs. ELA). Remarkably, this abnormality was effectively reversed in animals treated with PBM (Male, *P* < 0.0001, ELA+PBM vs. ELA; Female, *P* < 0.0001, ELA+PBM vs. ELA). Furthermore, higher co-localization of 4HNE Olig2 was also observed in ELA exposed animals, which was not evident in the PBM-treated animal group **(Figure 8A (h) and B (h))**.

**Figure 9:**
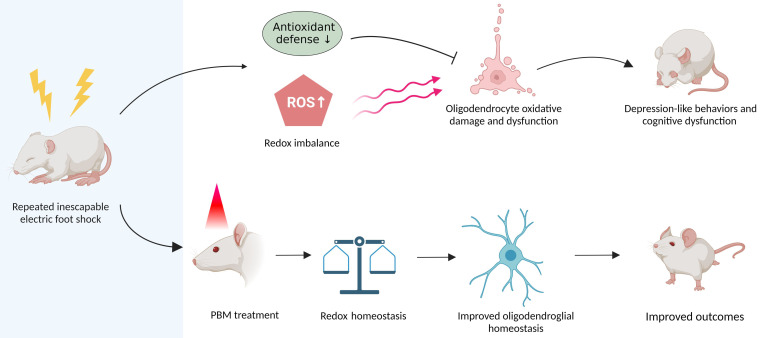
** Graphical abstract.** Repeated unpredictable electric foot shocks early in life lead to compromised antioxidant defenses and accumulation of ROS in the brain, which in turn causes oligodendrocyte oxidative damage and dysfunction as well as neurologic syndromes in adulthood. Conversely, early transcranial PBM treatment improves redox homeostasis in the brain, therefore helps maintain oligodendroglial homeostasis, resulting in better outcomes in animals.

Using fluorescent probe DHE, we next measured the level of ROS in the hippocampus. Consistently, animals exposed to ELA exhibited elevated ROS levels (Male, *P* < 0.0001, Control vs. ELA; Female, *P* < 0.0001, Control vs. ELA), and early PBM treatment suppressed this alteration (Male, *P* < 0.0001, ELA+PBM vs. ELA; Female, *P* < 0.0001, ELA+PBM vs. ELA). Furthermore, using the antioxidant kit, we further the total antioxidant capacity in the animals. As quantified in **Figure 9C (a, b)**, a compromised total antioxidant capacity was observed in ELA-exposed animals (Male, *P* = 0.0050, Control vs. ELA; Female, *P* = 0.0020, Control vs. ELA), which could be reversed by early PBM intervention (Male, *P* = 0.0175, ELA+PBM vs. ELA; Female, *P* =0.0192, ELA+PBM vs. ELA).

### Early PBM treatment does affect behavioral outcomes in normal animals

Our previous study demonstrated that PBM has no effect on normal animals 68. Moreover, a recent study measured the impact of PBM (810 nm laser, continuous wave, from postnatal day 24 to day 28) on the hippocampus of healthy rat pups, and no significant metabolic alternations were observed 78. To explore if early PBM treatment can influence postnatal development and behavioral outcomes in normal animals, we also examined weight change of animals treated with PBM alone. In comparison of the control group and those treated with PBM, there was no difference in the body weight alternation (**Figure S3A**). As shown in **Figure S3B and C**, compared to the control group, animals with early PBM treatment (from postnatal day 27 to day 33, last for 1 week) did not exhibit alternations in anxious and depressive-like behaviors. Also, no obvious change in cognitive performance was observed between the groups** (Figure S4A-B)**, as evidenced by the comparable discrimination index in the novel object recognition test and similar learning performances in the Barnes maze task.

## Discussion

In 1998, a landmark study by Felitti et al. pointed out the strong relationship between adverse childhood experiences and ill health conditions later in life 79. Since then, increasing attention has been given to investigating the long-term effects of ELA. It is now well recognized that ELA is linked to increased risk for negative health outcomes, including mental illness, cognitive disorder, and a wide range of chronic diseases 80-82. Remarkably, ELA has been associated with nearly 40% of depression cases in North America, and more than a quarter of depression and anxiety in Europe, according to a work published in *Lancet Public Health* 19. Also, the interaction between cognitive development, dementia risk, and ELA is now gaining increasing attention 83-85. Furthermore, the annual financial cost attributed to ELA was estimated to be US$581 billion in Europe and $748 billion in North America, respectively. 19. Unfortunately, effective early intervention for ELA is still inadequate, and novel treatments to prevent or alleviate the consequences of ELA are urgently needed.

Several rodent models of ELA have been developed, including the limited bedding/nesting conditions, maternal separation/deprivation, and chronic early stress, which simulate aberrant maternal care, early resource scarcity and child physical abuse, respectively 86, 87. To investigate the adverse consequences resulting from early traumatic stress, a previously established rodent model of ELA was employed in this study 64, 65. Using a battery of behavioral tests including the open field test, elevated plus test, Y maze and Morris water maze test, previous investigations have demonstrated that this ELA model leads to cognitive impairment, anxiety-and depressive-like behaviors later in adulthood. Consistent with these results, we found that exposure to repeated inescapable electric foot shock induced depressive-like symptoms as well as cognitive disorder later in adulthood. Furthermore, several significant sex differences have been observed. Specifically, although animals of both sexes demonstrated impaired recognition memory, the male animals exhibited more pronounced spatial learning deficits, suggesting males and females may exhibit different susceptibilities to ELA. These results were in line with previous findings that male animals exhibit higher vulnerability to cognitive impairment following ELA 88. Further studies, however, are necessary to elucidate the mechanisms underlying such sex differences.

The hippocampus is a crucial brain structure for cognitive function, emotional processing, and regulation. OLs in the hippocampus are sensitive to stress, and compromised OLs in the hippocampus contribute to cognitive deficits and mental disorders like depression 42, 89. Additionally, chronic stress has been reported to diminish the volumes of myelin sheaths and axons in the hippocampal CA1 region 90. Therefore, we focused specifically on the hippocampus, particularly the CA1 subregion. We found that pups subjected to foot shock exhibited abnormalities in oligodendrocyte lineage cells, which further led to myelin dysfunction. More importantly, we demonstrated for the first time that early PBM treatment (1st day after the last foot shock, 7 consecutive days) is sufficient to reverse these pathological alternations and prevent adverse neurological consequences in adulthood. Based on these findings, we proposed that PBM may represent a novel management strategy to reduce the adverse consequences of ELA.

PBM is a non-invasive technique that utilizes low levels of red and near-infrared light (wavelength between 600-1000 nm) to activate cellular activity 91, 92. Shortly after the discovery of lasers in the 1960s, it was recognized that laser therapy had the potential to ameliorate wound healing and reduce pain, and inflammation 57. These light therapies were soon applied to human patients and led to the concept of low-level laser irradiation therapy/photobiomodulation. The potential of brain PMB therapy has been supported by extensive research accumulated over the past decades, and its safety and therapeutic parameters have been practiced in preclinical animal studies and clinical trials 93. Therefore, brain PBM therapy is now considered a novel therapy for stimulating neural activity to improve brain function and has been introduced to the treatment of a variety of brain diseases, including depression, cognitive disorders, and dementia 46, 94, 95. Notably, in a rat model of post-traumatic stress disorder, previous work from our group demonstrated that early transcranial PBM administration was beneficial in attenuating stress event-induced comorbidities such as cognitive decline and depressive-like illnesses 48. Based on these findings, we sought to explore whether early PBM treatment could prevent similar neurologic sequelae resulting from ELA. Intriguingly, here we report that early treatment with PBM effectively prevented the depressive and anxiety-like symptoms exhibited in adulthood in animals exposed to ELA. Furthermore, this treatment protected against subsequent cognitive dysfunction, as evidenced by better performance on cognitive tests. Additionally, given that previous studies have suggested that ELA may reduce weight gain 71, we also investigated changes in weight gain in pups exposed to ELA, however, animals exposed to foot shock did not show a persistent reduction in body weight gain. Conversely, only a transient decrease in body weight gain was observed in the first week after ELA exposure in animals, which could be reversed by early PBM intervention. These findings may imply that early traumatic experiences do not have a far-reaching effect on weight variation. Taken together, these findings support the validity of PBM as an early intervention for ELA.

The multi-stage process of oligodendrocyte differentiation involves the transition of cells from immature, non-functional precursor cells to intermediately developed oligodendrocytes, and eventually to adult mature myelinating cells 96. Under normal physiological conditions, oligodendrocyte precursor cells (OPCs) continue to proliferate and generate myelinating oligodendrocytes. Myelination is often inhibited or delayed due to the failure of OPCs to differentiate into myelinating OLs 97, 98. The compromised physiological process of OPCs or oligodendrocyte maturation leads to the loss of myelin sheaths, which is also associated with depression, cognitive dysfunction and anxiety phenotypes 37, 38, 40. In the current study, although no substantial changes in the expression of OPCs were observed, ELA strongly ablated the ability of OPCs to differentiate into OLs. Intriguingly, this deficiency can be effectively prevented by early PBM treatment.

Myelin is a crucial evolutionary acquisition and adaptation, which supports the development of complex nervous systems in all vertebrates 99, 100. Myelin plasticity has emerged as a potential modulator of neuronal circuits, and the process of new myelin sheath formation, or myelination, is facilitated by certain neuroglia such as oligodendrocytes and schwann cells 101, 102. In the central nervous system, oligodendrocytes differentiated from OPCs produce the myelin sheath wrapped around the neuronal axons, whereas Schwann cells myelinating axons in the peripheral nervous system 102. Myelination not only enables the rapid and efficient transmission of electric impulses along the axon but also regulates axonal metabolism and neural plasticity 100, 103. Under normal conditions, myelination occurs throughout the life course and plays a critical role in the regulation of cognitive and other behavioral functions 104. Recent experiments from animals have informed us that oligodendrocytes dysfunction can lead to a variety of pathological processes, including depression, cognitive disorder, Alzheimer's disease, multiple sclerosis and other neurological conditions 26, 30, 42, 105. Intriguingly, a prior study has shown that early maternal separation (an animal model of early life adversity) resulted in a precocious the deficit of OPC pool and myelin loss in adults 43. More importantly, in a human autopsy study involving subjects with the history of child abuse, marked reduction immature OL cells has been observed 106, and those who experienced ELA also demonstrated reduced myelin growth across adolescence and young adulthood 107. Along the same lines, we found that early exposure to foot shock decreased the differentiation of OPCs into OLs, diminished proliferating OLs, and was accompanied by higher rate of apoptosis of OLs. Similarly, loss of myelinating oligodendrocytes was also observed, as indicated by lower MBP protein expression.

Endogenous and exogenous ROS continuously attack organisms, yet robust antioxidant defense mechanisms retain the balance between pro-oxidants and anti-oxidants in a stable redox state and preserve the body's homeostasis 108, 109. The imbalance between oxidative and antioxidant capacity can contribute to oxidative damage and lead to various illnesses 110. An increasing amount of evidence indicates that oxidative metabolism could be disrupted in response to stress, accompanied by toxic oxidation byproducts 111, 112. Stressful events in early life have been proven to induce long-term oxidative damage in adolescents and adult 113-115. Of note, due to lower levels of antioxidant enzymes and free radical scavengers, oligodendrocytes are sensitive to oxidative stress. Specifically, oligodendrocyte progenitor cells (OPCs) are more vulnerable to ROS injury than mature oligodendrocytes 75, 77, 116. Indeed, oxidative stress induced by oxidizing agents has been reported to disrupt the normal differentiation and proliferation processes of oligodendrocyte lineage cells 77. Therefore, we measured the level of oxidative damage and the total antioxidant capacity in animals. Intriguingly, we found that animals exposed to the ELA exhibited higher levels of ROS, as well as compromised antioxidant capacity. More importantly, oxidative damage in oligodendrocyte lineage was observed in ELA animals, as evidenced by double staining of 4HNE with PDGFRa or Olig2. Remarkably, early PBM treatment largely reversed these abnormalities. These findings suggest that ELA leads to redox imbalance and ROS accumulated in the hippocampus, which may underlie oligodendrocyte dysfunction in adulthood. Furthermore, early PBM treatment mitigates ELA-induced oxidative stress, which might provide a favorable microenvironment for the normal physiological processes within oligodendrocytes. Specifically, animals treated with PBM immediately after foot shock exhibited normal OPC differentiation and increased proliferation of OLs, as well as decreased apoptosis rates, compared to untreated animals. However, compared to animals exposed to ELA, PBM treatment did not significantly restore myelinating oligodendrocytes. A possible reason for this is that the one-week PBM treatment might not be sufficient to initiate the complete recovery of oligodendrocyte homeostasis, which subsequently prevented partial oligodendrocyte lineage cells from successfully converting to myelinating oligodendrocytes. This may also explain why greater improvement in spacial memory was not observed. These results emphasize the therapeutic promise of PBM, a non-invasive therapy, despite the incomplete recovery of oligodendrocyte homeostasis in the animals. To this end, further studies are needed to investigate the effects of extended treatment cycles and various doses.

Our study also contains some limitations. Firstly, we found that application of PBM early after ELA prevented the cognitive dysfunction and depression-like behaviors exhibited in adulthood, however, it is unclear whether this non-invasive therapy is helpful only when applied early after adverse experiences. Realistically, children who experience ELA often struggle to receive timely attention, which may lead to limited early treatment. Therefore, prior to clinical application, it is desirable to further explore whether later PBM administration can be equally effective in providing the beneficial effects. Additionally, validation of the optimal parameters for its application will need to be determined in various treatment windows after ELA. Moreover, in this study, the entire brains of the animals were exposed to PBM. In light of that PBM may bring distinct molecular effects when applied in different brain regions, therefore future studies will also be required to identify the best target brain regions for this therapy.

In conclusion, our study demonstrated that ELA resulted in oligodendrocyte dysfunction and long-term adverse neurological consequences, including depressive and anxiety-like behaviors, along with cognitive impairment in adulthood. Remarkably, early administration of PBM, a non-invasive procedure, largely prevented these deficits. Furthermore, these studies demonstrated that PBM attenuated the accumulated ROS and compromised antioxidant defense induced by ELA, which may underlie the beneficial effects of PBM. Taken together, these findings may provide a novel therapeutic strategy to prevent adverse neurological consequences resulting from ELA. From a translational perspective, further investigation is needed to determine the optimal treatment window and parameter for this treatment.

## Supplementary Material

Supplementary figures and table.

## Figures and Tables

**Figure 1:**
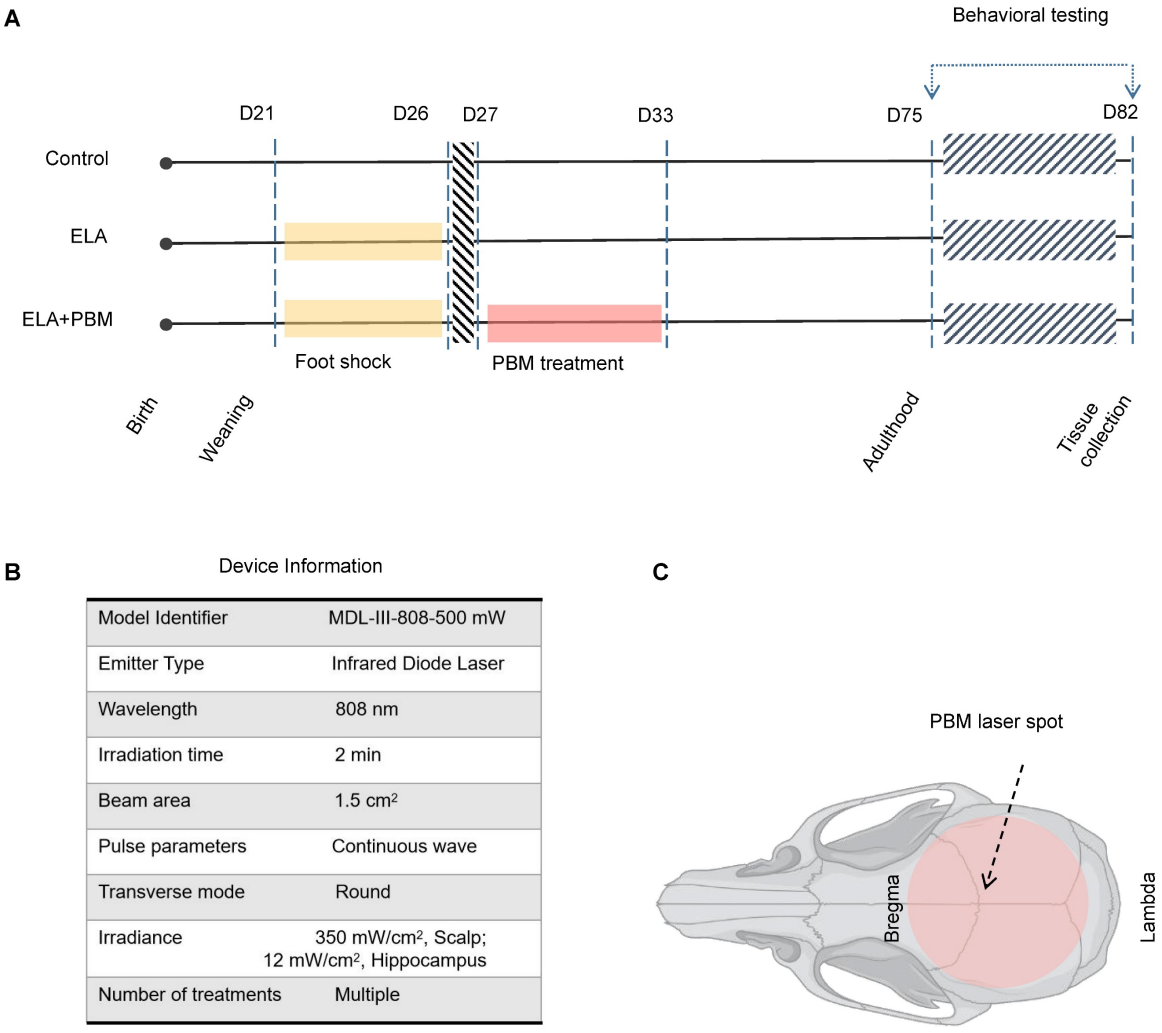
** Schematic diagram of the experimental protocol and device Information. A** Animals were randomly divided into 3 groups: (1) healthy animals without ELA (Control); (2) animals that received foot shock and sham PBM treatment (ELA); (3) animals that received foot shock with PBM treatment (ELA + PBM). PBM intervention was performed on the 1st day after the last foot shock and lasted for 7 days. Behavioral tests were performed from P75 to P81, followed by brain tissue collection. **B** Device information. **C** Red circle shadow denotes the area of PBM application on the rat skull.

**Figure 7:**
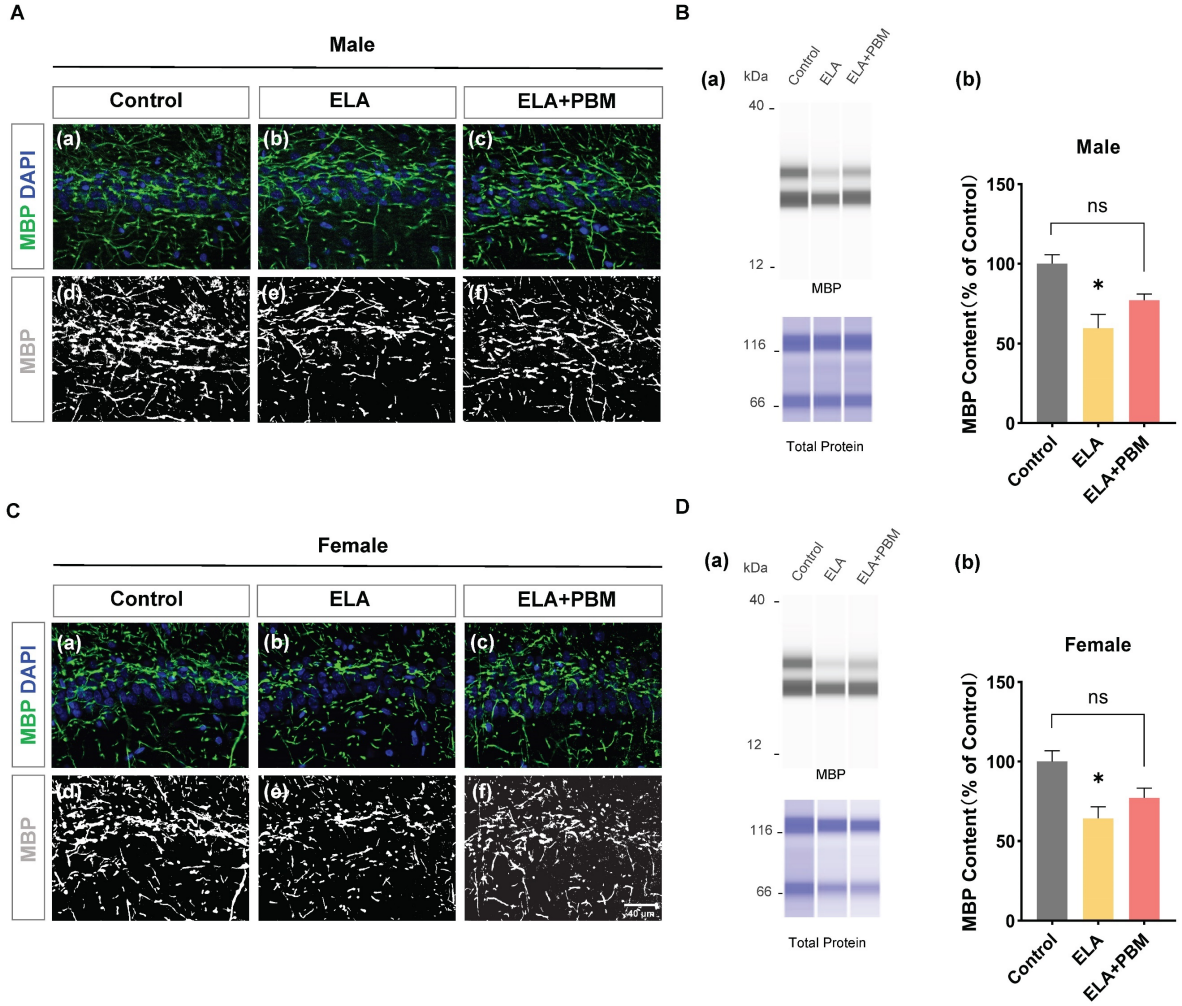
** ELA leads to reduction in myelinating oligodendrocyte, which can be partially mitigateded by early PBM treatment. A (a-c) and C (a-c)** Representative immunofluorescence staining for MBP in CA1 region of the hippocampus. **A (d-f) and C (d-f)** Representative images of MBP staining intensities were presented for a better view. **B (a) and D (a)** Representative synthetic protein bands of MBP in the hippocampus, corresponding peak areas were normalized with the level of total protein. ELA-exposed rats exhibited a marked reduction in MBP protein content, which was partially ameliorated by early PBM treatment. Scale bar = 40 µm. All data are presented as mean ± SE (n = 5-7). * P < 0.05 versus Control-group; ^#^ P < 0.05 versus ELA-group.

## Abbreviations

BL: Baseline
CCO: Cytochrome c oxidase
DHE: Dihydroergotamine
ELA: Early life adversity
OPCs: Oligodendrocyte progenitor cells
OLs: Oligodendrocyte lineage cells
PBM: Photobiomodulation
ROS: Reactive oxygen species
4HNE: 4-Hydroxynonenal
