# Supplementary material for: Photobiomodulation attenuates oligodendrocyte dysfunction and prevents adverse neurological consequences in a rat model of early life adversity
Source: Theranostics. 2023 Jan 16;13(3):913–30. doi: 10.7150/thno.78777 (PMC9925323; doi:10.7150/thno.78777)
Supplement: Supplementary file 1 — Supplementary figures and table. [file thnov13p0913s1.pdf]

Supplementary Information

4 Supplementary Figures

1 Supplementary Table

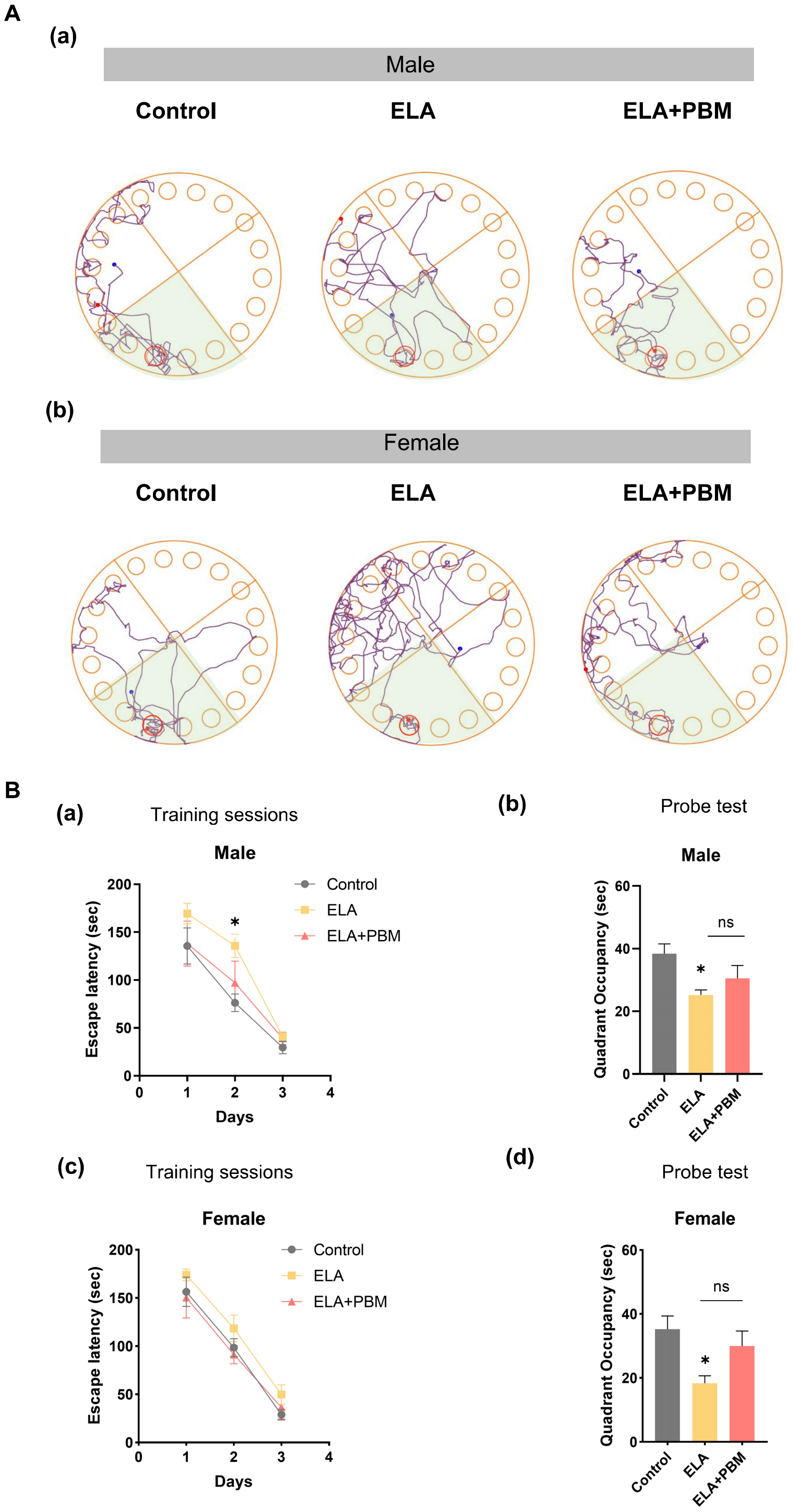
A
(a)
Male
Control
ELA
ELA+PBM
(b)
Female
Control
ELA
ELA+PBM
B
(a)
Training sessions
Male
Escape latency (sec)
Days
Control
ELA
ELA+PBM
*
(b)
Probe test
Male
Quadrant Occupancy (sec)
ns
*
Control
ELA
ELA+PBM
(c)
Training sessions
Female
Escape latency (sec)
Days
Control
ELA
ELA+PBM
(d)
Probe test
Female
Quadrant Occupancy (sec)
ns
*
Control
ELA
ELA+PBM

Figure S1. ELA resulted in spatial memory impairment, which can be partially alleviated by early PBM treatment. The Barnes maze task was used to assess spatial learning and memory abilities. **A (a, b)** The representative tracking plots of the Barnes maze task. **B** After training sessions, the probe trials were conducted, escape latency curves during the three days of training sessions **(a, c)** and quadrant occupancy time in the probe **(b, d)** were recorded and analyzed. Compared to control rats, ELA-exposed rats spent significantly less time in the target quadrant, and male rats also showed a deficit in spatial learning, early PBM treatment partially alleviated these deficits, but was not significant. All data are presented as mean ± SE (n = 5-7). * $P < 0.05$ versus Control-group; $^{\#}$ $P < 0.05$ versus ELA-group.

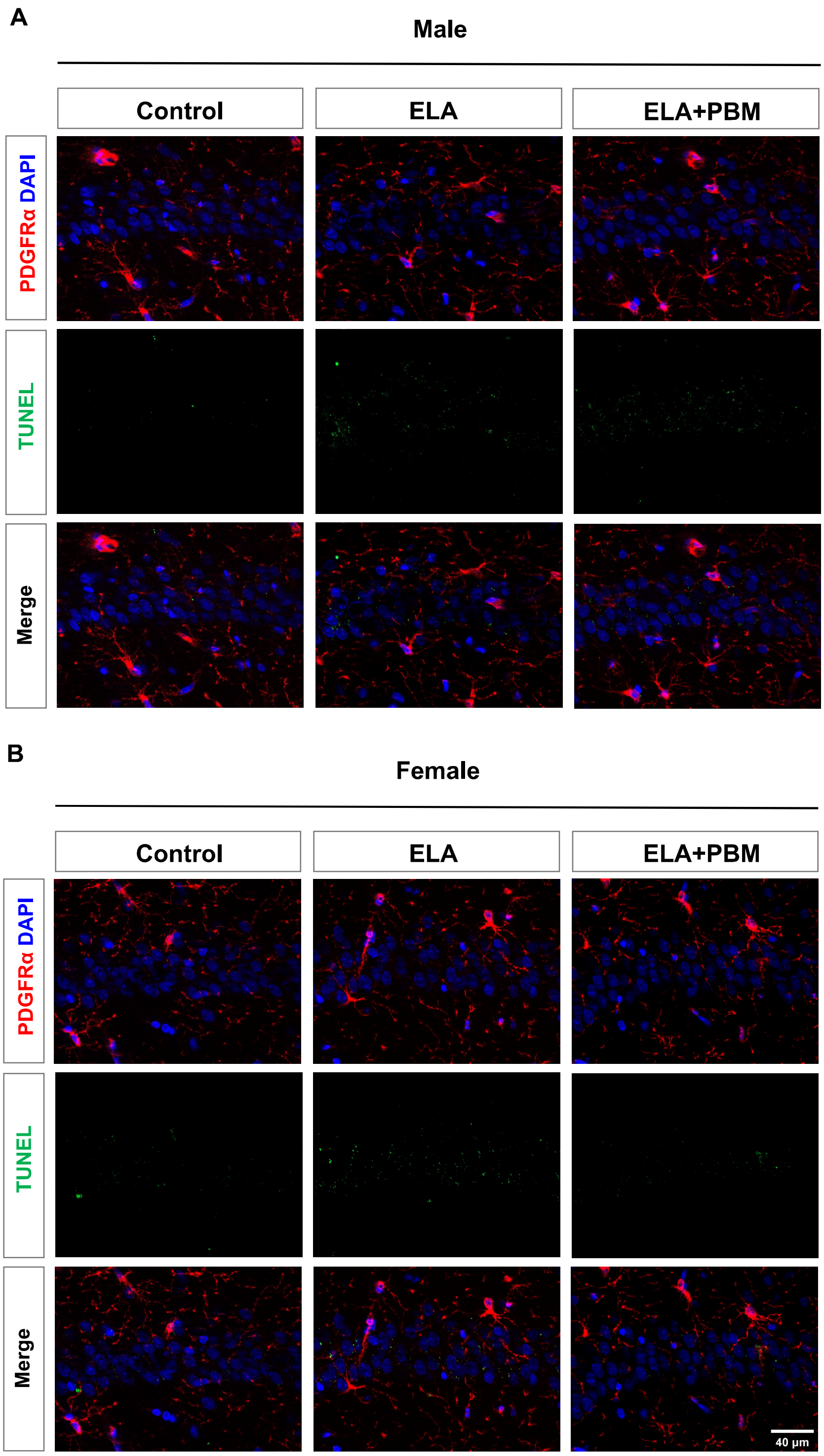
A
Male
Control
ELA
ELA+PBM
PDGFRα DAPI
TUNEL
Merge
B
Female
Control
ELA
ELA+PBM
PDGFRα DAPI
TUNEL
Merge
40 μm

Figure S2. ELA does not compromise the survival of the OPCs. TUNEL staining and immunostaining for PDGFRα (a marker of OPCs) with DAPI. In both male **(A)** and female animals **(B)**, no appreciable $Tunel^+$ $PDGFR\alpha^+$ cells were observed. Scale bar = 40 μm (n = 5-7).

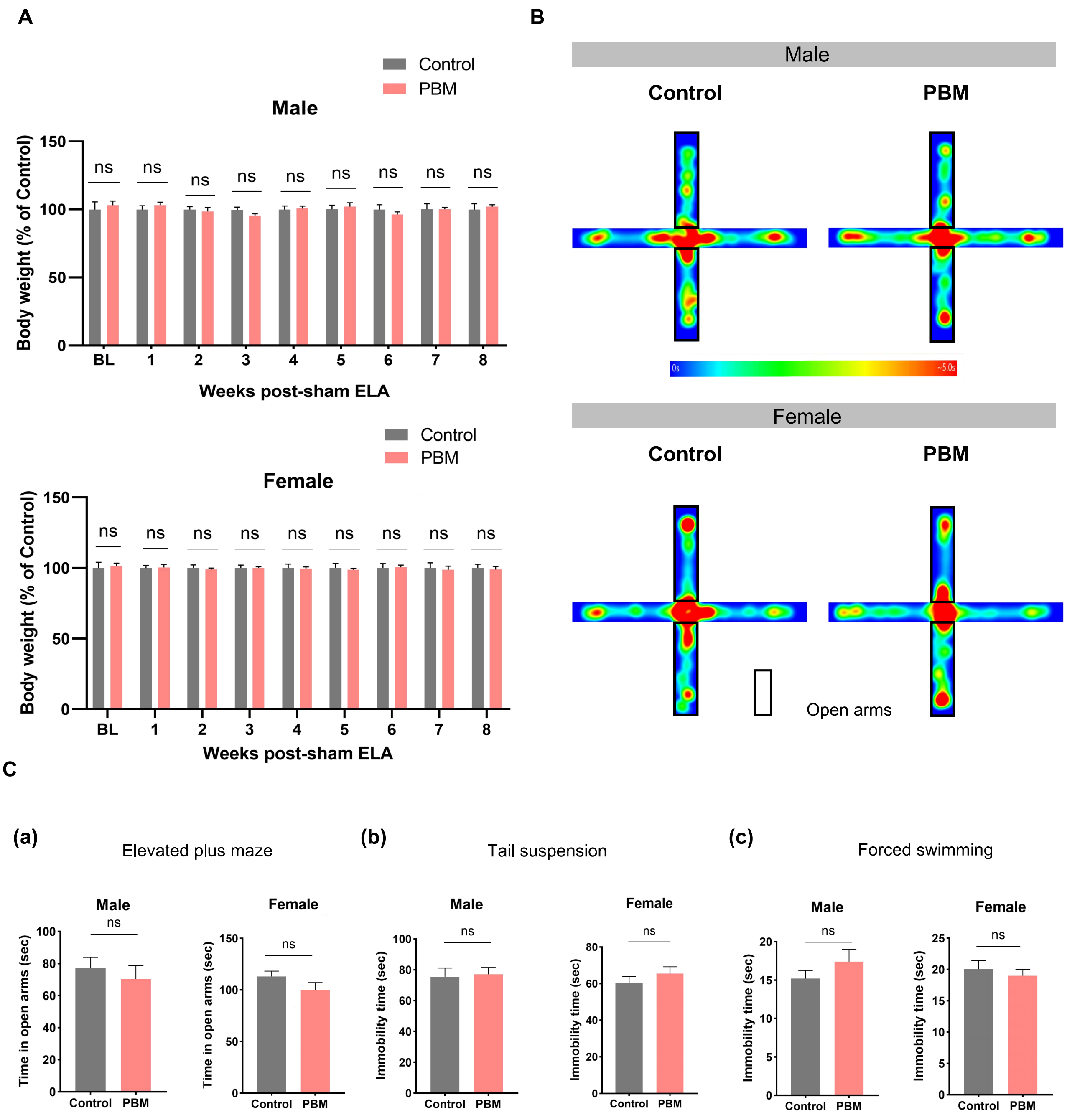
A
Control
PBM
Male
Body weight (% of Control)
150
100
50
0
ns
BL
1
2
3
4
5
6
7
8
Weeks post-sham ELA
Female
B
Male
Control
PBM
0s
~5.0s
Female
Open arms
C
(a)
Elevated plus maze
(b)
Tail suspension
(c)
Forced swimming
Male
Female
Time in open arms (sec)
Immobility time (sec)
Control
PBM

Figure S3. Early PBM treatment does not affect the levels of anxiety and depression in normal animals. A No significant difference in body weight alternation between the two groups. **B** Representative heat maps of the elevated plus maze test. **C (a)** There was no difference between groups in the time spent in open arms. **C (b)** No differences in immobility time were observed in the tail suspension test. **C (c)** No differences in immobility time were observed in the forced swimming test. All data are presented as mean ± SE (n = 6-7).

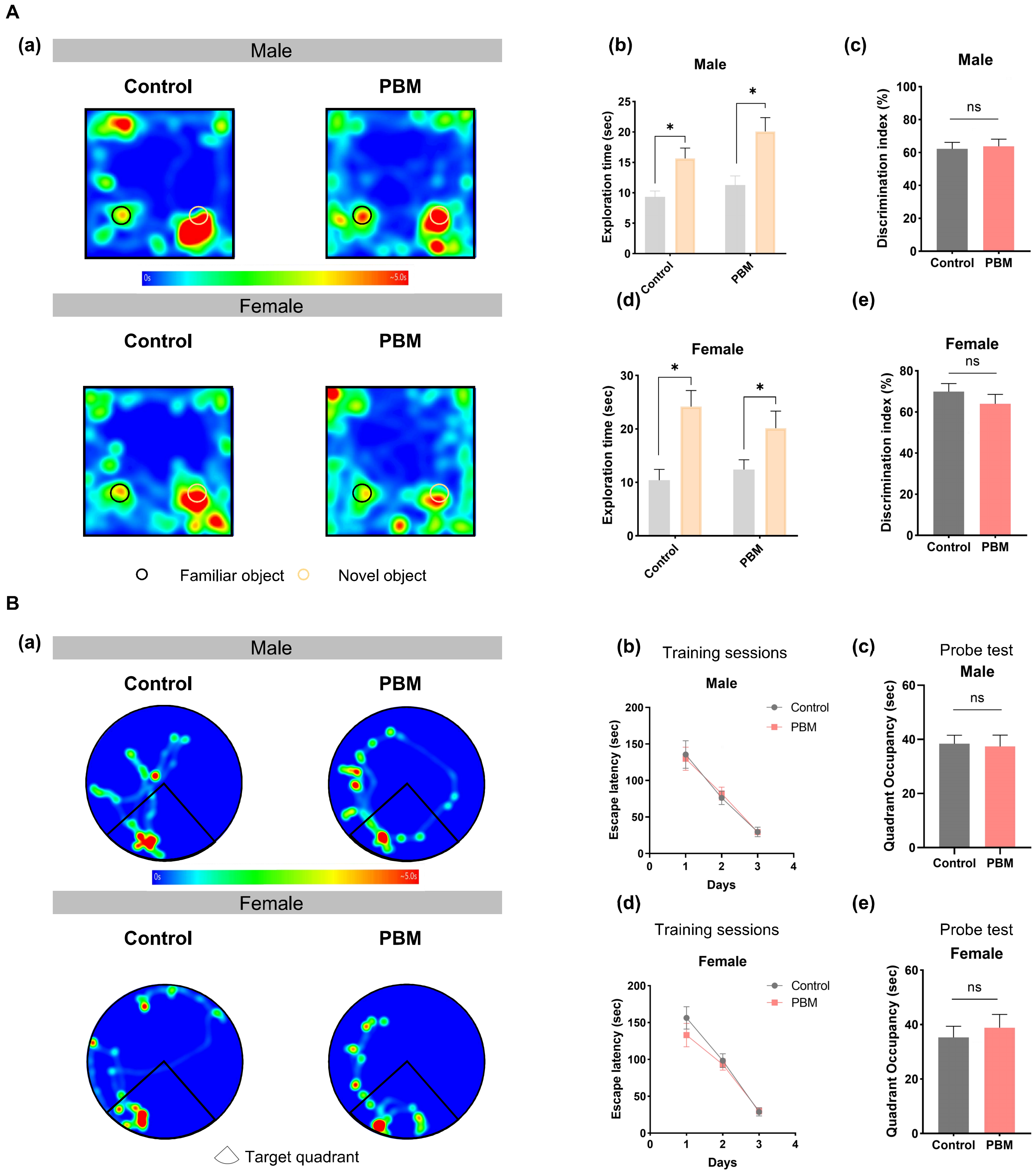

A
(a)
Male
Control
PBM
0s
~5.0s
Female
Control
PBM
Familiar object
Novel object
(b)
Male
Exploration time (sec)
25
20
15
10
5
0
*
*
Control
PBM
(c)
Male
Discrimination index (%)
100
80
60
40
20
0
ns
Control
PBM
(d)
Female
Exploration time (sec)
30
20
10
0
*
*
Control
PBM
(e)
Female
Discrimination index (%)
80
60
40
20
0
ns
Control
PBM
B
(a)
Male
Control
PBM
0s
~5.0s
Female
Control
PBM
Target quadrant
(b)
Training sessions
Male
Escape latency (sec)
200
150
100
50
0
Control
PBM
0
1
2
3
4
Days
(c)
Probe test
Male
Quadrant Occupancy (sec)
60
40
20
0
ns
Control
PBM
(d)
Training sessions
Female
Escape latency (sec)
200
150
100
50
0
Control
PBM
0
1
2
3
4
Days
(e)
Probe test
Female
Quadrant Occupancy (sec)
60
40
20
0
ns
Control
PBM

Figure S4. Early PBM treatment does not affect cognitive performances in normal animals. A (a) Representative heat maps of the novel object recognition test. **A (b, d)** The time spent on the familiar (dark) object and novel object (orange) was calculated and statistically compared. **A (c, e)** No differences in the discrimination index were observed between the two groups. **B (a)** Representative heat maps of the Barnes maze task. **B (b, d)** There was no significant difference in escape latency between the two groups. **B (c, e)** No significant differences in the quadrant occupancy time were observed between the two groups. All data are presented as mean $\pm$ SE (n = 6-7).

Table S1. Primary antibodies used in this study

Antibody	Host organism	Vendor	Cat#	Dilution	Application
Olig2	Goat	R&D Systems	AF2418-SP	1:250	IF
MBP	Mouse	Abcam	ab62631	1:300	IF
CC1	Mouse	EMD Millipore	OP80	1:500	IF
Ki67	Rabbit	Thermo Fisher Scientific	PA5-19462	1:300	IF
4-HNE	Mouse	Japan Institute For the Control of Aging	MHN-100P	1:200	IF
PDGFRα	Rabbit	Cell Signaling Technology	#3174	1:500 for IF, 1:50 for ProteinSimple®	IF, ProteinSimple® electrophoresis syetem
MBP	Rabbit	Proteintech	10458-1-AP	1:50	ProteinSimple® electrophoresis syetem
Olig2	Rabbit	Abcam	ab109186	1:50	ProteinSimple® electrophoresis syetem
